# Paediatric traumatic cataracts in Southwest China: epidemiological profile

**DOI:** 10.1186/s12886-022-02435-6

**Published:** 2022-05-06

**Authors:** Pingping Wang, Qingyu Fu, Hongbo Yin, Lin Wang, Longqian Liu

**Affiliations:** 1 Department of Ophthalmology, West China Hospital, Sichuan University, Chengdu, 61000 Sichuan China; 2 Department of Optometry and Visual Sciences, West China Hospital, Sichuan University, Chengdu, 61000 Sichuan China; 3 Laboratory of Optometry and Visual Sciences, West China Hospital, Sichuan University, Chengdu, 61000 Sichuan China; 4 The Department of Optometry and Visual Sciences, West China Hospital of Sichuan University, No. 37, Guoxue Lane, Chengdu City, Sichuan Province 610041 People’s Republic of China

**Keywords:** Paediatric traumatic cataract, Southwest China, Epidemiology, Infectious endophthalmitis

## Abstract

**Background:**

Paediatric traumatic cataracts are an important but preventable cause of acquired blindness. Understanding the epidemiology of paediatric traumatic cataracts is a prerequisite for prevention. This study aimed to characterize the epidemiological profile of paediatric traumatic cataracts in southwest China.

**Methods:**

The medical records of children (age range, 0–14 years old) who developed traumatic cataracts following open-globe injuries and were hospitalized at the Department of Ophthalmology at West China Hospital, between January 2011 and December 2020 were retrospectively analyzed. The demographic data, causes of injuries, posttraumatic complications, and visual acuity were recorded and analysed.

**Results:**

A total of 716 eyes from 716 patients were analysed in this study, including 521 (72.8%) males and 195 females in a gender ratio of 2.67:1; 117 of the patients were of ethnic minorities. Paediatric traumatic cataracts occurred more frequently in winter (32.5%). Sharp metal objects (scissors/knives/needles/sheet metal/nails/darts) – induced ocular injuries accounted for the highest proportion, followed by botanical sticks (wooden sticks /bamboo sticks /bamboo skewers)-induced injuries, and then stationery items (pencils/pens/rulers/paper)-induced injuries. The majority (68.7%) of the patients were aged 2–8 years, and the peak range of age was 4 - 6 years. The injuries were a result of penetrating trauma in 64.9% of patients, and blunt force trauma in the remainder (35.1%). Additionally, 131 (18.3%) cases developed posttraumatic infectious endophthalmitis after injuries. Patients with eye injuries caused by needles (*P* < 0.001), wooden sticks (*P* = 0.016), and bamboo skewers (*P* = 0.002) were at a greater risk of developing infectious endophthalmitis. The most common identified foreign organism was *Streptococcus*, which accounted for 42% (21/50) of all culture-positive specimens and was sensitive to vancomycin. Among the children who were younger than 5 years, 44.4% (55/124) of those with traumatic cataracts presented a corrected distance visual acuity less than or equal to 0.1 after undergoing cataract surgery, but among the children who were older than 5 years, this proportion was significantly smaller, just 20.4% of children aged 6-10 years (*P* < 0.001) and 18.4% of children aged 11-14 years (*P <* 0.001).

**Conclusion:**

The main causative agents of paediatric traumatic cataracts in southwest China were sharp metal objects, botanical sticks, and stationery items. Specific preventive measures are essential to reduce the incidence of paediatric traumatic cataract.

## Background

Ocular injuries are the most common cause of acquired blindness in developing countries, especially within the school-aged paediatric population; it is a major aetiology for cataract formation in normal-sized eyes [1–3]. Despite great advances in diagnostic and treatment methods, managing unilateral traumatic cataracts in visually immature children is a major challenge with typically unsatisfactory results, and sometimes result in permanent visual impairment. However, ocular trauma in children is more preventable than that in adult, and 90% of paediatric ocular traumas are preventable [4]. Most acute ocular injuries in children present a low risk of vision loss [5, 6]. However, ocular injuries in which the lenses are damaged remain a significant source of preventable blindness [6]. Therefore, in regards to the pediatric traumatic cataracts, effective methods of prevention are more important than surgical treatment. To prevent such serious conditions, evidence-based information on the causes of these injuries, public education and preventive measures are necessary. Studies on paediatric ocular trauma have been performed in the east [7], south [8], and south-central [9] parts of China, as well as other countries, such as Egypt [10] and the United States [11]. However, the aetiology and epidemiological characteristics often vary along with the socioeconomic status [12]. Southwest China is a relatively economically undeveloped area, and home to more than 30 ethnic minority groups. Therefore, it is hypothesized that the preventive measures based on the epidemiological characteristics of southwest China is likely different from those in other regions.

Infectious endophthalmitis is one of the most devastating ocular complications of open-eye trauma [13], and may lead to irreversible blindness in the infected eye within hours or days of symptom onset. Some causative agents put patients at a greater risk of developing infectious endophthalmitis [14]. Recognizing the causative agents putting patients at a greater risk of developing infectious endophthalmitis may motivate the public to seek medical attention earlier after suffering eye injuries, which may improve the treatment outcomes and even reduce the incidence rate of infectious endophthalmitis.

The Department of Ophthalmology at West China Hospital of Sichuan University (Chengdu, China), is one of the leading eye centres in China and the largest centre of its kind in southwest China. The demographic characteristics of paediatric patients with ocular injuries at the hospital are likely representative of the general situation in Sichuan, and even that in Southwest China.

## Methods

### Data collection and grouping

The medical records of children (ages, 0–14 years old) who developed traumatic cataracts following open-globe injuries and were hospitalized at the Department of Ophthalmology at West China Hospital, between January 2011 and December 2020, were retrospectively analyzed. The inclusion criteria were as follows: 1) patients aged 0–14 years at the time of getting injuries and 2) patients with open-globe injuries and visually significant traumatic cataracts. The exclusion criteria were as follows: 1) patients with preexisting ocular diseases and 2) patients with an originally poor visual acuity. Data included patients’ demographic characteristics, the objects causing the injuries, the type and location of the wounds, the posttraumatic complications, the organisms identified in endophthalmitis, results of sensitivity test, as well as corrected distance visual acuity (CDVA) at the final follow-up visit after undergoing cataract surgery. An international visual acuity measurement standard was used to examine the CDVA of the children who were older than 3 years old at the final follow-up visit, and the decimal notation of visual acuity was utilized for subsequent analyses. Only children with implanted intraocular lenses were selected for further analysis of visual acuity. Some patients refused to undergo intraocular lens implantation due to serious traumatic complications. The present study was performed in line with the principles of the Declaration of Helsinki, and it was approved by the Ethics Committee of West China Hospital of Sichuan University (Approval No. 2020.955). All data were retrospectively collected. The requirement of written informed consent was waived.

For the convenient display of the causative agents, some causative agents with similar characteristics were classified into one group. Objects such as scissors, knives, needles, nails, darts, and sheet metal were included together in one group of sharp metal objects, and fine wire and syringe needle were regarded as needles; pencils, pens, rulers, and paper were classified together as one group of stationery items; and wooden stick, bamboo stick, and bamboo skewer were included in the botanical stick group. According to the Birmingham Eye Trauma Terminology [15], open-globe injuries can be differentiated into two classes: the first class includes penetrating trauma caused by sharp objects, such as scissors, knives, and needles; the second class includes blunt force trauma caused by fists, hits, falls, fireworks explosions, balls, etc. Depending on the Open Trauma Score (OTS) [16], eye injuries can be classified into three zones on the basis of the wound location: Zone I includes injuries that the open wounds of the eyeball are isolated to the cornea or corneoscleral limbus; Zone II includes injuries that the wound involve the anterior 5 mm of the sclera; and Zone III includes injuries in which full-thickness wounds extend into the sclera more than 5 mm posterior to the corneoscleral limbus.

Patients with one or more of the following symptoms were diagnosed with endophthalmitis in the present study: hypopyon, an obviously cloudy vitreous, obscured retinal vessels, areas of necrosis of the retina, and positive culture of the intraocular fluid.

### Statistical analysis

Statistical analysis was performed using SPSS 21.0 software (IBM Corp., Armonk, NY, USA). The median and mean values were recorded for continuous variables. Parametric tests were used for analysing normally distributed continuous variables; nonparametric tests were utilized for analysing abnormally distributed continuous variables. The number of cases and percentages were recorded for categorical variables. The chi-square test was used to compare the differences when the number of cases was greater than 5; Fisher’s exact test was utilized to compare differences when the number of cases was less than 5. To determine the strength of association, the odds ratio (OR) was used with its 95% confidence interval (95% CI). Risk factors for endophthalmitis were studied by initially analyzing with univariate analysis, followed by multivariate logistic regression analysis of factors with *P* < 0.05. *P* ≤ 0.05 was considered statistically significant.

## Results

### Baseline data

A total of 716 patients (age range, 0-14 years) were enrolled in this study, including 521 (72.8%) male patients and 195 female patients in a gender ratio of 2.67:1. All traumatic cataract cases were unilateral and needed cataract surgery. Of these, 117 patients were members of ethnic minority groups, including 76 patients of Tibetan ethnicity, 37 patients of Yi ethnicity, 3 patients of Qiang ethnicity, and 1 patient of Bouyei ethnicity.

### Distribution of children in different years

**Fig. 1:**
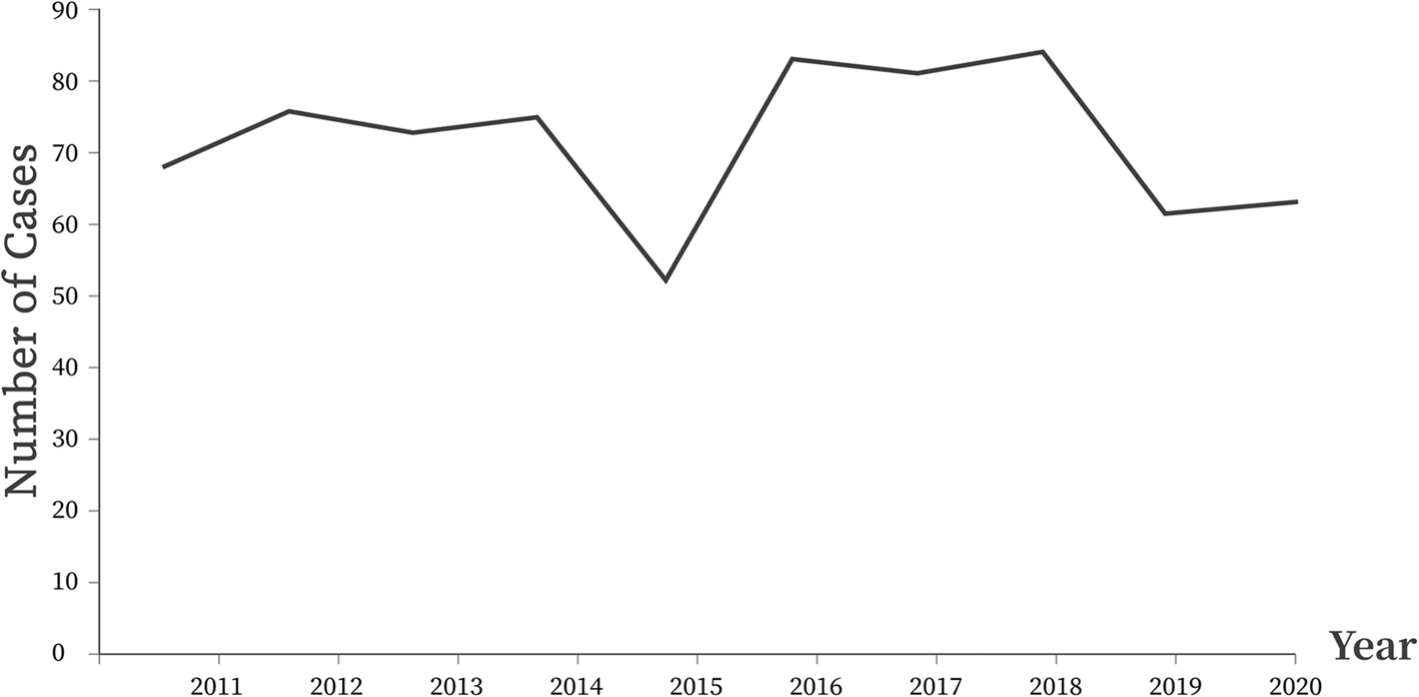
A line chart showing the number of cases of paediatric traumatic cataracts in different years

The number of cases of paediatric traumatic cataract patients in different years is shown in Fig. 1. In general, the number of cases of paediatric traumatic cataract showed a downward trend, while the tendency was not obvious. A significant drop was observed in 2015, followed by an immediate rise in 2016. The annual mean incidence of hospitalization for paediatric traumatic cataracts in West China Hospital of Sichuan University was estimated to be 5.89 per million (95% CI: 5.21- 6.57 cases per million).

### Distribution of children with traumatic cataract by age

**Fig. 2:**
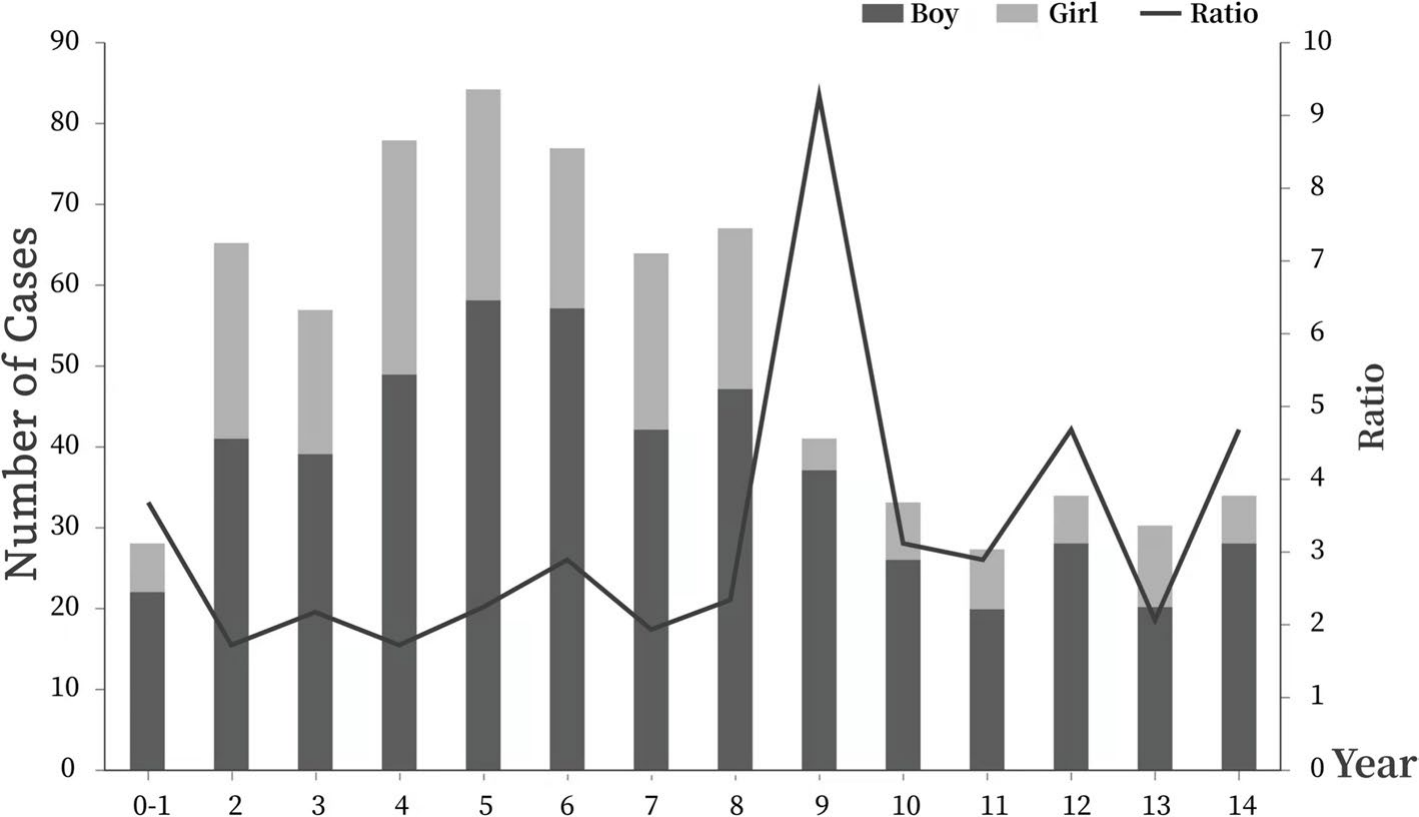
A bar chart showing the number of cases of traumatic cataracts in boys and girls in different age-based groups, and the line chart showing the ratios of boys to girls suffering traumatic cataract in different age-based groups

As shown in Fig. 2, the number of cases of paediatric traumatic cataract increased with age up until the age of 5 years, and decreased from the age of 6 years, peaking at the age of 5 years. The number of cases of decline was most noticeable at the age of 8 years. The majority (68.7%) of the patients were aged 2–8 years. The proportion of boys was higher than that of girls in each age-based group, and the ratio of boys to girls was relatively higher after the age of 8 years, peaking at the age of 9 years .

### Causes of ocular injuries

Table 1 lists the causes of the ocular injuries identified in the patients. The main causes were sharp metal objects (30.4%), botanical sticks (16.8%), and stationery (10.9%) items. Among those cases involving sharp metal objects, scissors involved in the greatest number of cases, accounting for (112/218) 51.4% of these cases. Wooden sticks were most frequently involved among the ocular injuries involving objects belonging to the group of botanical sticks, accounting for 78 of 120 such injuries (65%), and among injuries caused by wooden sticks, branches caused the majority of the injuries, accounting for 43 of the 78 injuries (55.1%). Pencils were the leading causative agent among the injuries caused by stationery item (57.7%, 45/78). Furthermore, 30 children were unable to recall the specific causes of their injuries, of whom 19 children suffered a strike from something, and 11 children were stabbed by something.

**Table 1:** Causes of ocular injuries leading to traumatic cataract

Cause of Ocular Injury	Number (n)	All (n)	Percentage
**Sharp metal objects (scissors/knife/needle/sheet metal/nail/dart)**	112/54/26/14/7/5	218	30.4%
**Botanical Sticks (wooden sticks/bamboo sticks/ bamboo skewer)**	78/15/27	120	16.8%
**Stationery items(pencil/pen/ruler/paper)**	45/20/9/4	78	10.9%
**Glass/ceramic**	43/8	51	7.1%
**Toy (plastic toy/bullet/slingshot/marble)**	27/4/7/2	40	5.6%
**Firecracker**	37	37	5.2%
**Hit**	29	29	4.1%
**Fall**	24	24	3.4%
**Stone**	20	20	2.5%
**Iron rod**	19	19	2.4%
**Light explosion**	11	11	1.5%
**Drying pole**	10	10	1.4%
**Animal**	8	8	1.1%
**Ball/ball bat**	3/5	8	1.1%
**Fist**	6	6	0.8%
**Clothing items**	3	3	0.4%
**Car accident**	3	3	0.4%
**Sport shoes**	1	1	0.14%
**Other**	30	30	4.2%
**All**	716	716	100%

### Variation of causes among age-based groups and years

Sharp metal objects and botanical sticks were the main causative agents of traumatic cataracts in children who were aged up to 6 years in both girls and boys. Among older age groups, significant variations were found in the distribution of the main causes between boys and girls. Botanical sticks accounted for a greater proportion of causative agents in boys, while stationery items accounted for a greater proportion of causative agents in girls. The three main causative agents of traumatic cataracts in boys and girls in different age-based groups are listed in Table 2.

**Table 2:** The top three causative agents of traumatic cataract in different age-based groups

Gender	Boys			Girls		
Age	Primary	Secondary	Tertiary	Primary	Secondary	Tertiary
**1-2**	Sharp metal objects	Glass	Botanical sticks	Sharp metal objects	Botanical sticks	Fall
**3-4**	Sharp metal objects	Botanical sticks	Firecracker	Sharp metal objects	Botanical sticks	Fall
**5-6**	Sharp metal objects	Botanical sticks	Stationery items	Sharp metal objects	Botanical sticks	Stationery items
**7-8**	Stationery items	Sharp metal objects	Botanical sticks	Sharp metal objects	Toy	Stationery items
**9-10**	Botanical sticks	Sharp metal objects	Stationery items	Sharp metal objects	Toy	Stationery items
**11-12**	Botanical sticks	Stationery items	Firecracker	Stationery items	Glass	Sharp metal objects
**13-14**	Sharp metal objects	Firecracker	Stationery items	Stationery items	Sharp metal objects	Botanical Sticks

**Fig. 3:**
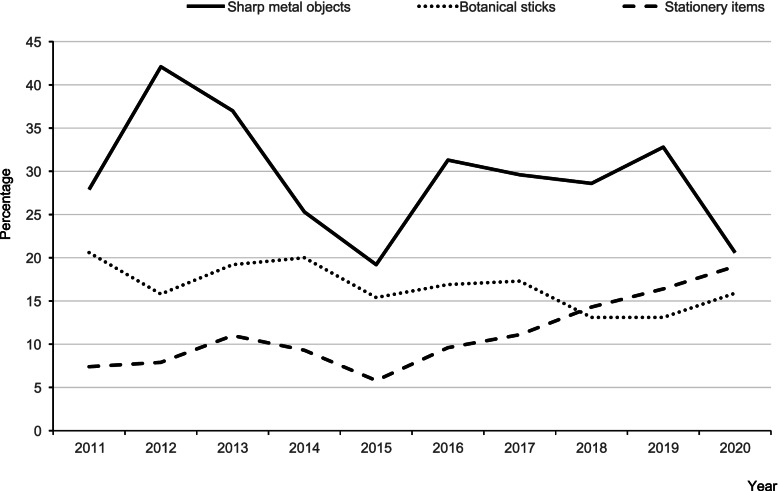
A line chart showing the percentage of the three leading causative agents in different years

In the current study, sharp metal objects were the leading cause of paediatric cataracts every year, followed by botanical sticks and stationery items, without a great change in the distribution of the three main causes over 10 years. Figure 3 illustrates the variation in the incidence rates of injuries caused by three main objects in different years. In recent years, injuries caused by sharp metal objects and botanical sticks have shown a downward trend, whereas injuries caused by stationery items have displayed an upward trend.

### Distribution of children with traumatic cataracts by season

As shown in Table 3, paediatric traumatic cataracts were more likely to occur in winter compared to any other seasons (32.5%) (*P* = 0.017, rank-sum test). However, there was no statistically significant difference in the number of cases which occurred in the in spring, summer, and autumn (*P* = 0.219, rank-sum test).

**Table 3:** Distribution of patients according to different seasons

Season	Spring (March-May)	Summer (June-August)	Autumn (September-November)	Winter (December-February)
Year				
**2011**	6	12	16	22
**2012**	22	21	15	22
**2013**	23	13	9	23
**2014**	24	15	16	25
**2015**	7	5	14	28
**2016**	26	11	18	26
**2017**	24	21	13	15
**2018**	23	26	20	25
**2019**	19	10	9	13
**2020**	13	16	16	33
**Median**	22.5	14	15.5	25
**All**	187(26.1%)	150(20.9%)	146(20.4%)	233(32.5%)

### Complications associated with ocular trauma

The present study included 465 (64.9%) cases with penetrating injuries and 251 cases with blunt force rupture injuries. Table 4 shows the other complications associated with ocular trauma. Rupture had a significantly higher incidence in causing anterior chamber haemorrhage (*P* = 0.004, the chi-square test), iridodialysis (*P < 0.001*, the chi-square test), vitreous haemorrhage (*P = 0.002*, the chi-square test), and retinal detachment (*P < 0.001*, the chi-square test). No significant difference was found in the number of cases of intraocular foreign bodies (*P* = 0.107, the chi-square test), infectious endophthalmitis (*P* = 0.451, the chi-square test), or wound involving the sclera (Zones II and III) (*P = 0.537*, the chi-square test).

**Table 4:** Complications associated with trauma

Complications	Penetrating	Rate (%)	Rupture	Rate(%)	***P***
**Anterior chamber haemorrhage**	39	8.4	38	15.1	0.004
**Iridodialysis**	31	6.7	43	17.1	0.000
**Vitreous hemorrhage**	31	6.7	34	13.5	0.002
**Retinal detachment**	19	4.1	29	11.6	0.000
**Intraocular foreign body**	39	8.4	29	11.6	0.107
**Infectious endophthalmitis**	84	18.1	47	18.7	0.451
**Zone II or Zone III**	56	12.0	30	12.0	0.537

### Postoperative visual acuity in different age-based groups

As listed in Table 5, 37.0% (265/716) of the children with traumatic cataracts had intraocular lenses implanted and postoperative examination of visual acuity. 124 (40.1%,124/309) children who were 5 years or younger, 103 (32.0%,103/322) children who were aged 6-10 years, and 38 (44.7%,38/85) children who were older than 10 years were included. Among the children who were aged 0- 5 years, 44.4% (55/124) of those with traumatic cataracts presented with a CDVA less than or equal to 0.1, while this proportion was significantly smaller in children older than 5 years, 20.4% (*P* < 0.001, rank-sum test) in those aged 6-10 years and 18.4% in those aged 11-14 years (*P <* 0.001, the rank-sum test).

**Table 5:** Postoperative visual acuity

Postoperative visual acuity	≤5 years old	6-10 years old	11-14 years old
≤0.1	55(44.4%)	21(20.4%)	7(18.4%)
0.1 -0.3	27(21.8%)	27(26.2%)	11(28.9%)
>0.3	42(33.9%)	55(53.4%)	20(52.6%)
Total	124(100%)	103(100%)	38(100%)

### Causative agents and microbiological features for infectious endophthalmitis

A total of 131 cases were diagnosed with infectious endophthalmitis (95 boys and 36 girls). The median length of time from being injured to being admitted to the hospital was 40 hours (24 h, 96 h). However, among those who did not suffer infectious endophthalmitis, this interval of time was significantly shorter, with a median time of 24 hours (10 h,72 h) (*P* < 0.001, rank-sum test).

Table 6 shows the causative agents of cases which developed infectious endophthalmitis. The ORs for wooden sticks (*P = 0.016,* the chi-square test), bamboo skewers (*P = 0.002,* the chi-square test), and needles (*P <* 0.001, the chi-square test) were higher than 1, and the 95% CI of the ORs did not contain 1, indicating that ocular injuries caused by wooden sticks, bamboo skewers, and needles were more likely to develop infectious endophthalmitis. Among cases of infectious endophthalmitis, 5 cases were caused by syringe needles; the OR for the syringe needle was 3.277 (95% CI: 1.023–10.491) (*P* = 0.003, the chi-square test). Therefore, in the present study, the paediatric traumatic cataract caused by a syringe needle put patients at a greater risk of developing infectious endophthalmitis i. After performing the multivariate logistic regression analysis of significant causative factors (wooden sticks, bamboo skewers, and needles) and time to admission, we found that the time to admission was not a risk factor for infectious endophthalmitis (*P* = 0.231), while wooden sticks, bamboo skewers, and needles were noted as risk factors (Table 6).

**Table 6:** Univariate and multivariate logistic regression analyses of the causative factors for infectious endophthalmitis

	Univariate analysis			Multivariate- logistic regression analysis		
Cause	Case number	OR (odds ratio)	***P***	***OR***	***P***	95% CI
**Scissor/knife/needle/sheet metal**	18/6/14/2	0.83/0.54/5.71/0.74	0.51/0.16/0.00/0.76	–	–	–
**Wooden sticks/bamboo Skewer/bamboo sticks**	22/11/4	1.91/3.26/1.64	0.016/0.002/0.50	–	–	–
**Glass/ceramic**	4/2	0.44/1.50	0.12/0.64	–	–	–
**Iron rod**	4	1.20	0.76	–	–	–
**Animal**	3	2.72	0.17	–	–	–
**Fire cracker**	7	1.10	0.92	–	–	–
**Pencil/ pen/ruler**	12/2/3	1.69/0.49/2.26	0.13/0.40/0.38	–	–	–
**Fall**	3	0.63	0.60	–	–	–
**Hit**	2	0.32	0.11	–	–	–
**Stone**	2	0.49	0.40	–	–	–
**Sport shoes**	1	–	–	–	–	–
**Plastic toy**	5	1.01	1.00	–	–	–
**Light explosion**	1	0.44	0.50	–	–	–
**Other**	3	0.23	0.48	–	–	–
**Time(h)**	–	–	–	1.00	0.23	0.99-1.00
**Wooden sticks**	–	–	–	1.91	0.023	1.09-3.34
**Bamboo Skewer**	–	–	–	4.93	0.000	2.24-10.86
**Needle**	–	–	–	3.94	0.002	1.59-7.36

Among 131 cases who were diagnosed with infectious endophthalmitis, 86 had vitreous samples taken for bacterial culture and sensitivity tests. Cultural positivity was observed in 50 (58.1%) cases. A single bacterial species was isolated in 48 of these cases, and multiple organisms were isolated in 2 of these cases. The most common Gram-negative organism in the present study was *Streptococcus*, accounting for 42% (21/50) of the cases. The second most commonly found Gram-negative bacterial organism was *Staphylococcus* (6/50) and *Enterobacter* (6/50). Among bacteria within the Enterobacter species, five were *Enterobacter cloacae,* all of which were from cases caused by were wooden sticks. The majority of the organisms were sensitive to vancomycin.

## Discussion

The present study was motivated by the lack of data on paediatric ocular trauma in southwest China. The study aimed to obtain and summarize the aetiological and clinical characteristics of severe ocular injuries in children who were admitted to West China Hospital.

### Aetiological and epidemiological profiles

The number of cases of paediatric traumatic cataracts treated at West China Hospital decreased slightly. Hence, paediatric traumatic cataracts remained a major source of blindness. The sudden drop in the number of cases in 2015 might be partly because a sudden ban on fireworks was enforced. In all age-based groups, traumatic cataracts occurred predominantly in boys, which was consistent with previous findings reported in Shanghai (China )[7], Lithuania, [17] and Spain [18]. These findings could be explained by the more aggressive and violent nature of activities which boys were more likely to be involved in than girls. In addition, the ratio of boys to girls increased after the age of 8 years. The reason may be that the psychological maturity in boys lagged behind that in girls, and thus, boys were less aware of self-protection. Children aged 4–6 years old were at a higher risk of getting traumatic cataracts. This was possibly because children within this age group could walk independently, but their guardians may not always accompany them, resulting in insufficient supervision. In addition, children within this age group are typically curious and eager to explore their external environment, while simultaneously having a limited awareness of danger. Despite their inadequate self-protection ability, children eventually learn the concept of danger through their daily observations. Thus, the occurrence of ocular injuries could decline with age, as evidenced by the data. The noticeable decrease in the number of cases of traumatic cataracts observed in children who were older than 8 years may be attributed to schooling and homework. Regular presence at school could reduce their time in which children may participate in dangerous activities.

The causes of ocular injuries were diverse and tended to vary among different countries. This may be due to different socioeconomic backgrounds and living environments. The main causes of paediatric traumatic cataracts in the present study were sharp metal objects, followed by botanical sticks, and stationery items. The results of the present study were different from Yu et al.’s findings [7] on pediatric traumatic cataracts in Shanghai, which showed toys to be the second main cause; in other countries, such as in India, the main causes were wooden splinters, bows, and arrows [19, 20], while the main cause in Egypt was wooden sticks [10].

The main causative agents seem to vary according to the timing of children’s physical development. For boys and girls aged < 6 years, sharp metal objects were the leading cause, followed by botanical sticks. It was concluded that boys and girls before the age of 6 years were engaged insimilar daily activities [21], which also explained the relatively lower ratio of boys to girls in the number of cases of traumatic cataracts in the younger age groups.

In 2020, an outbreak of a new coronavirus infection occurred in China. Children spent more time at home with their parents, but the number of cases of paediatric traumatic cataracts increased. This phenomenon could be explained by the fact that paediatric traumatic cataracts occur most frequently at home, which has been confirmed by other studies. The home was the most common place where paediatric ocular injuries occurred, followed by outdoor places and schools [22, 23]. In addition, the most common causes in the present study were scissors and knives that are typically found in every household, which also supports the notion that the home may be the primary place ocular traumas occur. Therefore, it is necessary to emphasize the importance of preventive measures in the home environment. In addition, the decreasing contribution of sharp metal objects, and the increasing contribution of stationery items might have resulted from universalized compulsive education and increased attention to the dangers of sharp metal objects.

The results of the present study also pointed to a seasonal difference among the times of year that injuries most commonly occur. The majority of the injuries in the present study occurred in winter (32.5%). Other studies conducted in different areas reported that the injuries frequently occurred during summer in Wenzhou (China) [9] and Canada [24], and during autumn in southwestern Turkey [21]. These seasonal differences might result from the climatic characteristics and lifestyles of people in the regions where the studies were conducted. In Sichuan, China, where the present study was performed, the second longest holiday vacation period is in winter, in which children generally spend a lot of time playing, while parents are often busy preparing for holiday festivities. In addition, during this time firecrackers are set off as a traditional custom. These conditions accounted for the higher percentage of cases of ocular trauma which occurred during winter. The reason comparatively fewer injuries occurred in the spring and autumn seasons may likely stem from the fact that children were in schools and busy with homework during most of those times. Summer, which is quite hot in the region, may have limited some of the children’s activity.

### Microbiological profile of infectious endophthalmitis

The present study revealed that injuries caused by needles, wooden sticks, and bamboo skewers carried a higher risk of developing posttraumatic infectious endophthalmitis. This may be due to needles having greater levels contamination and the ability to more easily penetrate deeper into the eye, and potentially because the opening of the wounds are sometimes small, causing parents to be negligent of the injuries, resulting in delays in time before patients receive therapy. Wooden sticks, as organic matter, carry a greater risk of being contaminated [25]. For bamboo skewers, deeper penetration, ignorance, and easy contamination could explain the results.

The most commonly isolated organism related to posttraumatic infectious endophthalmitis in the present study was *Streptococcus*, which was consistent with the findings of Alfaro et al .[26] and Al-Rashaed et al .[27]. While the present study’s findings were different from those of Yan et al.’s systematic review on Chinese paediatric posttraumatic infectious endophthalmitis, who showed that the most common organism was *Staphylococcus epidermidis* [28]. In the present study, a more virulent species, *E. cloacae* [29], was involved in several cases. The isolated organism was reported to be a commensal organism of the gastrointestinal tract, rather than the ocular surface [30], and it can also be found in soil and plants. It therefore was no surprise that the cases in which *E. cloacae* was identified were caused by wooden sticks.

### Preventive measures

As for the prophylaxis of paediatric trauma, we should emphasize educational and legislative measures, such as informing parents, teachers, and children about the causative factors and potential hazards of ocular injuries, as well as restricting the availability of dangerous items to children through public service announcements. Parents and doctors should regularly study suggestions for eye protection. Some suggestions are as follows: (1) Younger children should be kept away from sharp metal objects and be paid extra attention when they use them. Tips of scissors for household should be blunted at the time of manufacturing. (2) As home is the main place where pediatric ocular traumas occur, children should be provided a safe home environment. For example, sharp metal objects should be kept out of the reach of children. (3) Children should be assisted at an earlier age to facilitate the development of a sense of what is safe and what is not through health education. (4) Children should wear protective spectacles when playing with toys, such as marble, slingshot and toy guns. (5) Laws should be implemented for manufacturers to inform consumers of potential dangers and to minimize the inherent risks associated with particular products by including child-resistant packaging, printed warnings, and age recommendations. Multicentre studies should be performed to garner support for such legislation. (6) The government should restrict the sale of firecrackers to underage children. Additionally, underage children should not set off firecrackers without permission from their parents or guardians and they should be taught to keep a safe distance when firecrackers are set off. (7) Boys and children aged 4–6 years old are at a higher risk of traumatic cataracts, and thus, they require further attention.

The present study had several limitations. First, it had some inherent biases because of its retrospective design. Second, the data were limited to patients’ medical records. Third, the study probably slightly underreported the actual incidence rate of pediatric traumatic cataract in the Sichuan Province because only injuries treated at West China Hospital were included. Thus, multicentre research is needed. Finally, only injuries associated with the open-globe traumatic cataract were included in this study. Close-globe injuries and those not involving the traumatic cataract were excluded. Therefore, this study cannot represent the epidemiological characteristics of other types of ocular trauma. In that regard, additional comprehensive research is needed.

## Conclusions

The epidemiological characteristics of paediatric traumatic cataracts, such as the main causative factors, were found to be highly dependent on the area of the population studied. In the southwest of China, the main causative factors of paediatric traumatic cataracts were sharp metal objects, botanical sticks, and stationery items, and the injuries occurred most frequently during winter. Injuries resulting from needles, wooden sticks, and bamboo skewers carried a higher risk of posttraumatic infectious endophthalmitis, and the most commonly isolated organism was *Streptococcus.* A better understanding of the epidemiological characteristics would be advantageous for implementing specific preventive measures.

## Data Availability

The data that support the findings of this study are not publicly available because sharing these data might compromise the privacy of the research participants, but the data are available from the corresponding author upon reasonable request.

## Abbreviations

CI: Confidence interval
OR: Odds ratio
CDVA: Corrected distance visual acuity
OTS: Open Trauma Score
